# Supplementary material for: Experimental realization of Lieb-Mattis plateau in a quantum spin chain
Source: Sci Rep. 2020 Jun 8;10:9193. doi: 10.1038/s41598-020-66336-8 (PMC7280303; doi:10.1038/s41598-020-66336-8)
Supplement: Supplementary file 1 — Supplementary Information. [file 41598_2020_66336_MOESM1_ESM.doc]

**Experimental realization of Lieb-Mattis plateau**

**in a quantum spin chain**

Hironori Yamaguchi*, Taiki Okita, Yoshiki Iwasaki, Yohei Kono, Nao Uemoto,

Yuko Hosokoshi, Takanori Kida, Takashi Kawakami, Akira Matsuo,

and Masayuki Hagiwara

*Author to whom correspondence should be addressed.

E-mail: yamaguchi@p.s.osakafu-u.ac.jp

**Crystal structure and interchain interactions**

The crystallographic data for (4-Br-*o*-MePy-V)FeCl_4_ are summarized in Table S1, and the molecular structure is shown in Fig. S1a. The verdazyl ring (which includes four N atoms), the upper two phenyl rings, and the bottom methylpyridyl ring are labeled *R*_1_, *R*_2_, *R*_3_, and *R*_4_, respectively. The dihedral angles of *R*_1_-*R*_2_, *R*_1_-*R*_3_, *R*_1_-*R*_4_ are approximately 33^◦^, 20^◦^, and 19^◦^, respectively. The results of the MO calculations indicate that approximately 61% of the total spin density is present on *R*_1_. Further, while *R*_2_ and *R*_3_ each account for approximately 18% and 17% of the relatively large total spin density, *R*_4_ accounts for less than 4% of the total spin density. Therefore, the intermolecular interactions are caused by the short contacts related to the *R*_1_, *R*_2_, and *R*_3_ rings. In this supplemental, we describe the exchange interactions between 4-Br-*o*-MePy-V molecules with spin-1/2, which correspond to the interactions between the mixed spin chains in the main text. The MO calculations indicated two types of dominant interactions. They are evaluated as *J*_1_/*k*_B_ = 7.0 K and *J*_2_/*k*_B_=-5.2 K. The molecular pair associated with *J*_1_ is related through an inversion center and has C-C short contacts of 3.45 Å, as shown in Fig. S1b. The molecular pair associated with *J*_2_ is also related through an inversion center and has N-C short contacts of 3.53 Å, as shown in Fig. S1c. The *J*_1_ and *J*_2_ form an alternating chain along the *a* axis, which is perpendicular to the mixed spin chain, and the mixed spin chains are partially connected by *J*_1_ and *J*_2_, as shown in Figs. S1d and S1e.

In the experimental low-temperature regions, the magnetic ground state on the mixed spin chain is stabilized, and the spins on 4-Br-*o*-MePy-V can be regarded as forming a nonmagnetic singlet dimer through *J*. The effective interactions between the mixed spin chains have exchange paths composed of *J*-*J*_1_-*J* or *J*-*J*_2_-*J* and are caused through the triplet excited states of the two singlet dimers. Accordingly, the effective interchain interactions become much weaker than the MO evaluations.

Table S1: Summary of crystallographic data for (4-Br-*o*-MePy-V)FeCl_4_.

| Compound | (4-Br-*o*-MePy-V)FeCl_4_ | |
| --- | --- | --- |
| Temperature (K) | 293(2) |  |
| Formula | C_20_H_18_BrCl_4_FeN_5_ |  |
| Crystal system | Monoclinic |  |
| Space group | *P*2_1_/*c* |  |
| Wavelength (Å) | 0.7107 | |
| *V*(Å^3^) | 2381(3) | |
| *a* (Å) | 7.676(7) |  |
| *b* (Å) | 19.425(15) |  |
| *c* (Å) | 16.028(13) |  |
| *β* (degrees) | 94.962(16) |  |
| *Z* | 4 |  |
| *D*_calc_(g cm^-3^) | 1.690 | |
| Total reflections | 4094 |  |
| Reflection used | 2630 |  |
| Parameters refined | 281 |  |
| *R* | 0.0451 |  |
| *R_w_* | 0.0994 |  |
| Goodness of fit | 1.050 |  |
| CCDC | 1967790 |  |
|  |  |  |


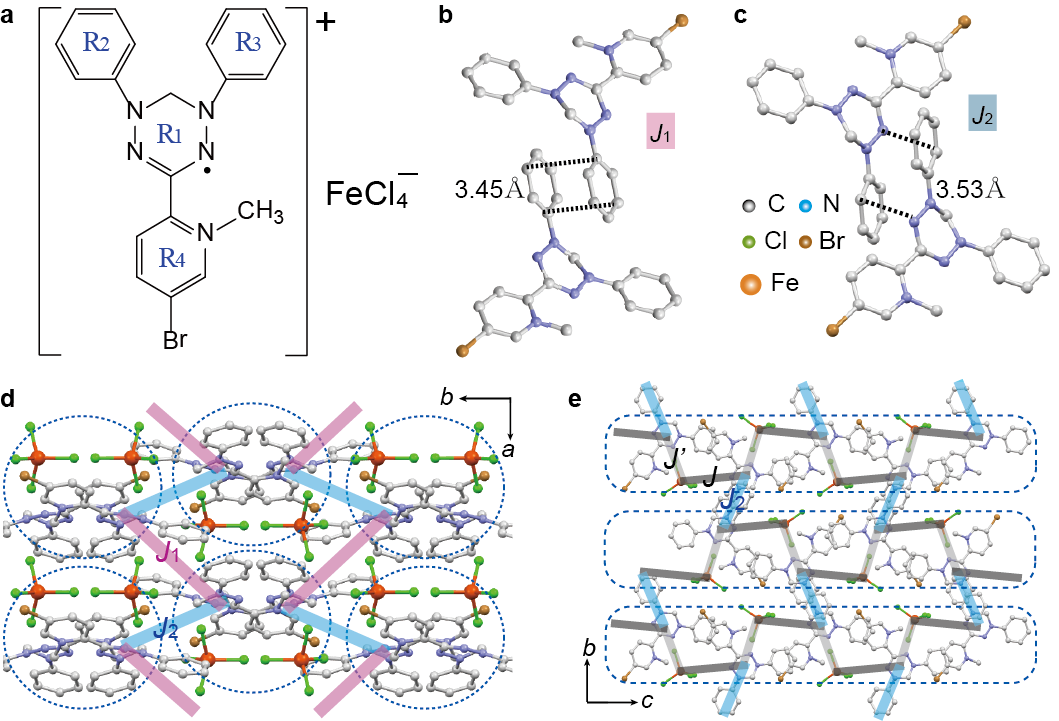


Figure S1: **a,** Molecular structures of (4-Br-*o*-MePy-V)FeCl_4_. Molecular pairs associated with exchange interactions (**b**) *J*_1_ and (**c**) *J*_2_. Hydrogen atoms are omitted for clarity. The broken lines indicate short contact related to the rings with high spin-density distributions. Crystal structure in the (**d**) *ab* and (**e**) *bc* planes. The broken line encloses molecules comprising each mixed spin chain structure. The thick solid lines represent the exchange couplings.
